# Collaborating by courier, imaging by mail

**DOI:** 10.15252/embr.201949755

**Published:** 2019-12-16

**Authors:** Rüdiger Schweigreiter, Christopher Cawthorne, Saskia Lippens, Geert Van Minnebruggen, Sebastian Munck

**Affiliations:** ^1^ Biocenter Institute of Neurobiochemistry Innsbruck Medical University Innsbruck Austria; ^2^ MoSAIC—Molecular Small Animal Imaging Centre KU Leuven Leuven Belgium; ^3^ VIB Bio Imaging Core and VIB‐UGent Center for Inflammation Research Ghent Belgium; ^4^ Department of Biomedical Molecular Biology Ghent University Ghent Belgium; ^5^ Flanders Institute for Biotechnology VIB Ghent Belgium; ^6^ VIB Bio Imaging Core and VIB‐KU Leuven Center for Brain & Disease Research Leuven Belgium; ^7^ Department for Neuroscience KU Leuven Leuven Belgium

**Keywords:** Methods & Resources, S&S: Ethics

## Abstract

Core facilities offer visiting scientists access to equipment and expertise to generate and analyze data. For some projects, it might however be more efficient to collaborate remotely by sending in samples.

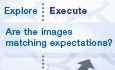

Many research institutes have established core facilities as a repository of technology and know‐how that provide scientists access to multiple techniques and data analysis. Imaging and microscopy along with sequencing and proteomics have been at the forefront of this development, and core facilities and their expertise have been a key factor for many successful research projects 1. One of the challenges for a core facility is building a competitive and sustainable portfolio along with access models so scientists can efficiently and easily use the services 2.

To promote access by external users, several high‐profile institutes—including the European Molecular Biology Laboratory (EMBL) and HHMI Janelia Research Campus as well as national/international initiatives such as Euro‐BioImaging and Global BioImaging—have established visitor programs to facilitate short‐term access to their infrastructure similar to large‐scale facilities such as synchrotrons or observatories. Researchers can visit the host institution for a period of a few weeks to carry out the necessary experiments at the core facility 3. Alternatively, the core facility could send equipment to the researcher if the laboratory has sufficient expertise 4, but this option is fairly limited, and not well suited for carrying out pilot experiments or explorative work.

While a short‐term visit (STV) allows visitors to perform complex experiments, the timescale poses limitations. Re‐implementing an experimental approach in a new environment is often tedious and challenging, as is adjusting and fine‐tuning workflows. It may also not be possible to schedule sophisticated *in vivo* experiments that require repetitive, often weekly interventions. In practice, the results from such visits are often more limited than hoped for. In addition, some practical hurdles may apply such as shipping vertebrate animals, which require the approval of an ethics committee. The STV model thus may not be optimal for open‐ended scientific projects or addressing more demanding research questions that go beyond routine experiments.

This is usually not a problem for sequencing and proteomics core facilities, which routinely receive samples from external users through couriers. It raises the question whether collaborations with a microscopy facility can be similarly established based on shipping samples rather than extended or repeated visits. Such a sample‐centered access model could help to establish and maintain longer‐term and open‐ended collaborations. With Europe currently investing in infrastructure for the purpose of increasing STVs, we feel that a wider discussion about expanding access models to imaging facilities is needed to ultimately benefit research infrastructure projects.

An example of a successful external collaboration based on shipping samples is the recently published method on imaging axon regeneration within synthetic nerve conduits 5. Imaging early‐stage nerve regeneration within optically challenging nerve guidance conduits promises to yield clinically relevant information on the efficacy of various treatment regimens. The respective scientific interests of the core facility and the external research group had been aligned, and clear expectations and milestones were formulated (the collaborating researchers were 748 km apart). The external user performed nerve surgery on mice, and the core facility carried out imaging and analysis on shipped samples. The project took more than 2 years, with samples being shipped in 2‐week periods. The partners interacted only remotely, making use of commonly available tools such as remote desktop solutions and team rooms for analyzing microscope data and manuscript writing.

Based on this experience 5, 6, we propose a gated process for interacting with external collaborators over distance (Fig 1). Of course, external collaborators can also come from nearby institutions. We argue that the preparation of biological samples at the source and shipping them to the imaging facility is often more efficient than re‐implementing the biological model at the facility. Also, sending samples means less traveling, less pressure on professional and private schedules, and a lower carbon footprint. Most importantly, a collaboration by courier improves the reproducibility of data and allows the completion of long‐term, open‐ended research projects.

**Figure 1:**
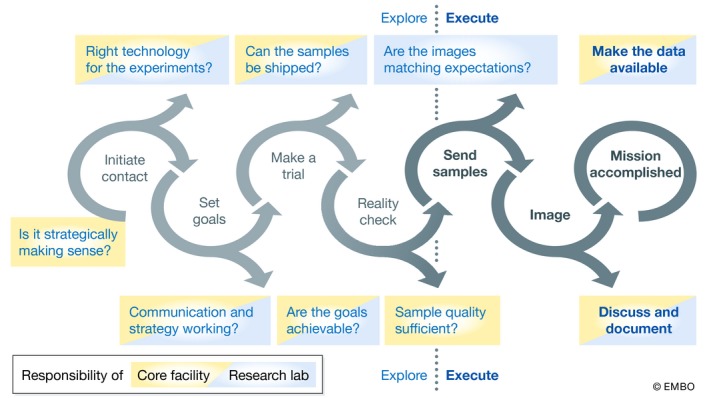
Flow diagram of the remote interaction of a core facility with an external collaborator

The underlying limitation is the nature of the sample and its preparation; however, we believe that optimizing shipping procedures is worth an investment of time and effort for the reasons outlined above. For us, shipping the nerves suspended in clearing liquids was a solution, but other modes can be envisioned: for instance, setting up spatial sequencing on fixed tissue sections, carrying out high content screening tasks for endpoint assays, or imaging whole‐mount samples on light‐sheet microscopes, to name only a few. In general, shipping is possible whenever the sample can be robustly prepared or if the project involves a general measurement in easily cultivatable cells or related models. In addition, it is possible to use specific biological models that exist at the research institute of the imaging partner.

Shipping samples, not scientists, has the potential to improve the delivery of projects with elevated research impact. External collaborators get access to critical infrastructure and expert knowledge, while the facility gains important expertise, which in turn helps to maintain its competitiveness. Shipping samples has the potential to enrich service portfolios, accelerate discoveries 7, and contribute to the facility's success 8, ultimately benefitting the host institution 9. It has the potential to become the most suitable access model for some imaging facilities, allowing them to carry out projects and long‐term strategic interactions that otherwise could not be done via STVs. In any case, the overall objective, regardless of the access model, should be to enable the best science.
